# Select controversies in the management of methicillin-resistant *Staphylococcus aureus* bacteremia: answers and remaining questions from recent evidence

**DOI:** 10.12703/r/10-66

**Published:** 2021-08-31

**Authors:** Jose F Suarez, Sharon Ong’uti, Marisa Holubar

**Affiliations:** 1 Jackson Memorial Hospital/University of Miami Miller School of Medicine, Division of Infectious Diseases, Miami, FL, USA; 2 Stanford University School of Medicine, Division of Infectious Diseases and Geographic Medicine, Stanford, CA, USA

**Keywords:** Methicillin resistance, Staphylococcus, *Staphylococcus aureus*, MRSA, bacteremia

## Abstract

Methicillin-resistant *Staphylococcus aureus* (MRSA) bacteremia continues to cause significant morbidity and mortality despite advances in medical therapy. Vancomycin therapy remains the standard of care for most cases of MRSA bacteremia but has pharmacokinetic and pharmacodynamic limitations, dosing complications, and known toxicity. Welcomed clinical trials have recently addressed some of the controversies that plague this field, including optimization of vancomycin dosing and use of combination therapy. In this review, we discuss these trials and their implications for clinical care and future research.

## Introduction

*Staphylococcus aureus* bacteremia (SAB) remains a distinct entity in the realm of infectious disease, singular in its ability to adhere to vascular structures, cause deep-seated infections, disseminate, and result in a high mortality despite targeted antibiotic therapy. In 2017, the Centers for Disease Control and Prevention estimated 120,000 cases of SAB in the US and 20,000 associated deaths. Of those cases, mortality was higher among those with methicillin-resistant *Staphylococcus aureus* (MRSA) bacteremia in comparison with their methicillin-susceptible counterparts^1^. MRSA bacteremia has been associated with longer hospitalizations, longer durations of bacteremia, more severe disease (as evidenced by higher Charlson comorbidity and Pitt bacteremia scores), and a higher 30-day mortality in comparison with methicillin-susceptible *Staphylococcus aureus* (MSSA) bacteremia^2^.

MRSA bacteremia poses a particular challenge aside from its increased mortality. The first-line therapy, vancomycin, exhibits variable penetration into tissues, slow killing, known nephrotoxicity, and complex dosing requirements^3^. In addition, clinicians face a dearth of evidence upon which to make management decisions for patients with MRSA bacteremia. However, in recent years, clinical trials addressing some of these controversial topics have examined vancomycin dosing optimization and the use of combination therapy. In this article, we review select clinical trials (in adults) published in the last three years and discuss their implications for clinical care and future research.

## Optimizing vancomycin dosing

The primary pharmacokinetic/pharmacodynamic (PK/PD) exposure target for all glycopeptide antibiotics, including vancomycin, is the ratio of the 24-hour area under the curve to the minimum inhibitory concentration (AUC/MIC). However, the AUC/MIC range that maximizes clinical efficacy while minimizing toxicity is unclear. In 2009, national guidelines recommended an AUC/MIC target of at least 400 mg*hour/L and, despite limited data, promoted the use of trough (Cmin) monitoring as a surrogate given the challenges of calculating an accurate AUC/MIC in routine practice^4,5^. These recommendations were rapidly accepted.

However, a growing number of studies have called both the AUC/MIC target of at least 400 mg*hour/L and the Cmin monitoring approach into question. Studies have reported increased nephrotoxicity with vancomycin AUC/MIC of at least 600 mg*hour/L^6^ despite being within the accepted target range of the 2009 national guidelines. This recommended target range was based on limited data from predominantly retrospective studies that suggested that a high AUC/MIC was associated with less clinical failure^7^.

The PROVIDE trial was designed to address this issue and test the hypothesis that patients with MRSA bacteremia would experience less frequent treatment failure if they received higher vancomycin exposure, defined as exceeding a set PK/PD threshold (AUC/MIC_BMD_ > 650 mg*hour/L or AUC/MIC_e-test_ > 320 mg*hour/L depending upon whether the MIC was determined by broth microdilution [BMD] or e-test). This multicenter observational study prospectively evaluated the association between vancomycin exposure (using day 2 AUC/MIC) and treatment failure, defined as 30-day mortality or persistent bacteremia of at least 7 days, in patients with MRSA bacteremia^8^. Of the 265 evaluable patients, 18% experienced treatment failure and 26% developed acute kidney injury (AKI). Treatment failure did not differ by high versus low vancomycin exposure (21% vs. 11%, *P* = 0.07), but high vancomycin exposure was associated with nephrotoxicity. Further analysis suggested an optimal AUC/MIC ceiling of 515 mg*hour/L to maximize clinical efficacy while limiting nephrotoxicity. Owing to lack of power, the study was not able to define a lower bound of a target range.

The narrower target AUC/MIC range suggested by that study may be challenging to achieve in routine clinical practice. Based in part on the PROVIDE trial data, U.S. 2020 national guidelines added a ceiling to the goal AUC/MIC range, recommending a target AUC/MIC of 400 to 600 mg*hour/L for MRSA bacteremia and other serious MRSA infections in adult patients^9^.

These guidelines also recommended replacing Cmin-based dosing with AUC-guided dosing and monitoring^9^. Retrospective and simulation studies demonstrate that many patients can achieve the target AUC/MIC with Cmin levels of less than 15 mg/L when an MRSA isolate’s vancomycin MIC is not more than 1 µg/mL, indicating a lack of correlation between a vancomycin Cmin level and AUC/MIC target^6,10^. Furthermore, Cmin of greater than 15 mg/L has been associated with nephrotoxicity, which indicates that Cmin-based dosing can lead to unnecessary and excessive vancomycin exposure and patient harm. In contrast, studies found that using AUC-guided dosing is associated with decreased nephrotoxicity, reduced per-patient blood sampling, and lower doses overall without impacting clinical outcomes^11,12^. However, it is important to note that robust data regarding the clinical efficacy of AUC-guided dosing are not yet available.

The 2020 national guidelines outline two methods to achieve AUC-guided drug monitoring: first-order PK equations and Bayesian software programs. In addition to the discussion included in these guidelines, AUC-guided dosing implementation options and considerations have recently been reviewed and individual institutions have published real-world experience^12–15^. Regardless of the method used, converting to an AUC-guided approach is often resource-intensive (financial, personnel time, and information technology enhancements) and may be most impactful if targeted at patients receiving more than 3 days of therapy to maximize value^13^. Areas of uncertainty include how to continue drug monitoring in patients who require vancomycin in the outpatient setting, where trough-based monitoring is most common and AUC-guided dosing would be logistically challenging. Further prospective studies are needed to refine AUC/MIC targetand evaluate practical ways to achieve these goals in routine practice.

Despite optimizing dosing strategies, vancomycin may not be the drug of choice for some patients. MRSA bacteremia due to strains with vancomycin MIC_BMD_ of greater than 1 µg/mL poses a particular challenge to vancomycin use, rendering the target AUC/MIC of 400 to 600 impossible to achieve with standard dosing. For these infections, the use of alternative agents is warranted^9^. Fortunately, despite local reports of the emergence of vancomycin resistance, MRSA isolates with vancomycin MIC of greater than 1 µg/mL remain uncommon in large epidemiologic studies^16–18^. Evidence also suggests that vancomycin MICs are often specific to the methodology used and that exact agreement between different methodologies is relatively uncommon^19^. For example, the e-test method consistently generates higher MICs than BMD, the reference standard^19^. Clinicians must work with their local laboratory to confirm and interpret MRSA isolates with vancomycin MIC of greater than 1 µg/mL.

## Combination therapy

*In vitro* data demonstrate and *in vivo* data suggest that combining bactericidal antibiotics with activity against MRSA, such as vancomycin or daptomycin, with beta-lactams (antibiotics to which the organism is inherently resistant) enhances pathogen elimination^20^. Some mechanisms by which beta-lactams could enhance vancomycin’s or daptomycin’s activity include the following: reduction of the bacterial cell wall thickness, which could facilitate target access for vancomycin or daptomycin^21^; an increase in the negative charge of the cell membrane, which would facilitate binding of daptomycin (which acquires a positive charge when it complexes with calcium)^22,23^; and the ability of beta-lactams to enhance production of antimicrobial peptides in the infected host, which could lead to enhanced bacterial killing^22,24^. Unfortunately, the theoretical advantage of combination therapy has not translated into consistent improvement of clinically significant outcomes in the real world. Indeed, recent prospective cohort studies and randomized controlled trials have yielded mixed results, and increased side effects and costs have been met with marginally meaningful clinical benefits in specific patient populations. A summary of some of these studies is provided in Table 1.

**Table 1.** Table of summary statistics from studies comparing standard of care with combination therapy in MRSA bacteremia management.

Study	Monotherapy	Combination therapy	*P* value
CAMERA-2 trial^27^			
Study design	Open-label, randomized clinical trial		
Sample size	n = 178	n = 174	
Intravenous line source	12%	14%	
Treatment	VAN or DAP	VAN or DAP + beta-lactam^a^	
Composite primary outcome^b^	39%	35%	0.42
All-cause mortality (day 42)	11%	15%	0.29
Persistent bacteremia (day 5)	20%	11%	0.02
Toxicity outcome: AKI	6%	23%	<0.001
Zasowski *et al*.^30^			
Study design	Retrospective cohort study		
Sample size	n = 129	n = 229	
Treatment	VAN	VAN + cefepime	
Endovascular source	15.5%	27.9%	0.008
Primary outcome: microbiologic failure^c^	25.3%	38.0%	0.012
30-day mortality	7.8%	20.5%	0.002
BSI ≥7 days	31.0%	18.8%	0.008
Vancomycin-associated nephrotoxicity^d^	5.4%	5.2%	0.94
Geriak *et al*.^29^			
Study design	Pilot, prospective randomized study		
Sample size	n = 21	n = 17	
Endovascular source	35%	47%	
Treatment	VAN or DAP	DAP + ceftaroline	
Treatment failure after 5 days, no. of patients^g^	3	1	
In-hospital mortality	26%	0%	0.029
Bacteremia duration, median days	3	3	0.56
AKI, no. of patients	1	0	
Jorgensen *et al*.^32^			
Study design	Retrospective, comparative cohort study		
Sample size	n = 157	n = 72	
Endovascular source	40.8%	31.9%	
Treatment	DAP	DAP + beta-lactam^e^	
Composite clinical failure^f^	27.4%	12.5%	0.013
30-day mortality	11.5%	6.9%	0.351
Persistent bacteremia at 5 days	31.7%	19.4%	0.078
AKI	2.9%	10.8%	0.046
*Clostridioides difficile* diarrhea	1.3%	5.6	0.08
McCreary *et al*.^33^			
Study design	Retrospective, multicenter, matched cohort study		
Sample size	n = 113	n = 58	
Endovascular source	53%	53%	
Treatment	VAN or DAP	DAP + ceftaroline	
Mortality within 30 days	14.2%	8.3%	>0.05
Median MRSA bacteremia duration (days)	4.8	9.3	<0.001
Bacteremia relapse/recurrence	9.7%	8.6%	NS
Truong *et al*.^31^			
Study design	Retrospective cohort study		
Sample size	n = 47	n = 63	
Treatment	VAN	VAN + beta-lactam^h^	
Endocarditis	17.0%	22.2%	0.631
Implantable cardioverter-defibrillator/cardiac device	2.1%	4.8%	0.634
Treatment failure^i^	41.9%	30.4%	0.291
MRSA-related inpatient mortality	11.1%	8.2%	0.740
Persistent bacteremia^j^	18.6%	19.3%	1.000
AKI^k^	19.2%	14.3%	0.604

AKI, acute kidney injury; BSI, blood stream infection; DAP, daptomycin; MRSA, methicillin-resistant *Staphylococcus aureus*; NS, not significant; VAN, vancomycin. ^a^Beta-lactam included flucloxacillin, cloxacillin, or cefazolin. ^b^Composite primary outcome, four components: all-cause mortality, persistent bacteremia at study day 5, microbiological relapse defined as a positive blood culture for MRSA at least 72 hours after a preceding negative culture, and microbiological treatment failure defined as a positive sterile site culture for MRSA at least 14 days after randomization ^c^Microbiologic failure, defined as a BSI duration of at least 7 days and/or MRSA BSI recurrence within 60 days of the end of MRSA BSI therapy ^d^Vancomycin-associated nephrotoxicity defined as a serum creatinine increase of 0.5 mg/L and 50% from baseline on two consecutive measurements from initial vancomycin dose to 72 hours after the last dose ^e^Beta-lactam included cefepime, cefazolin, ceftaroline, ceftriaxone, meropenem, piperacillin-tazobactam, ertapenem, and ampicillin-sulbactam. ^f^60-day mortality or 60-day recurrence or both ^g^Persistent MRSA bacteremia after 5 days ^h^Beta-lactam included piperacillin-tazobactam, ceftriaxone, ceftaroline, cefepime, and meropenem. ^i^Composite of clinical and microbiologic failure ^j^Defined as 5 days of positive MRSA blood cultures ^k^An increase in serum creatinine by 50% or 0.5 mg/dL, whichever was greater, from baseline in accordance with RIFLE (risk, injury, failure, loss, end-stage renal disease) criteria.

## Vancomycin combination therapy

Vancomycin-based combination therapies have been used in clinical practice to treat serious MRSA infections for many years; however, supportive evidence from prospective clinical trials was lacking^25^. The CAMERA trial, a small proof-of-concept study, found that vancomycin in combination with an anti-staphylococcal penicillin (flucloxacillin) shortened the duration of bacteremia by 1 day compared with vancomycin alone in patients with MRSA bacteremia^26^. This led to a larger, multicenter, open-label randomized controlled trial (CAMERA-2) that compared MRSA bacteremia patients who received vancomycin monotherapy (with a goal trough of 15–20 µg/L) or daptomycin (dosed at 6–10 mg/kg per day) with those who received a combination of vancomycin plus an anti-staphylococcal penicillin (flucloxacillin or cloxacillin) or cefazolin (used for those allergic to penicillin or receiving dialysis)^27^. Enrollment occurred 72 hours after a positive blood culture was obtained. The primary outcome was defined as a composite of 90-day mortality, persistent bacteremia at day 5, microbiological failure, and relapse.

Of the 174 patients who were randomly assigned to the combination group, 111 received flucloxacillin or cloxacillin (64%) and 27 received cefazolin (16%). Almost all of the 178 in the monotherapy arm received vancomycin alone (172, 97%). The trial was stopped early by the data safety monitoring board at 80% recruitment because of a statistically significant increase in AKI in the combination group (23% vs. 6%, 17.2% difference; 95% confidence interval [CI] 9.3–25.2%) and no difference in the composite primary outcome. Of note, at the time of study closure, fewer patients who received monotherapy cleared their bacteremia at day 5 compared with those who received combination therapy (11% vs. 20%, difference −8.9%, 95% CI −16.6 to −1.2%).

This robust study provides definitive evidence of nephrotoxicity with no clear evidence of clinical benefit when vancomycin combination therapy is used. However, broad generalizations are limited by the agents used in this study. First, only 3 (2%) of 174 patients in the combination group received daptomycin and no vancomycin; this is important as vancomycin itself is known to be nephrotoxic and daptomycin combination therapy has shown some promise in other studies^28,29^. Second, most patients in the combination group received an anti-staphylococcal penicillin, which is also associated with nephrotoxicity. Notably, of the 27 patients who received vancomycin together with cefazolin, only one experienced AKI, suggesting that beta-lactams other than semisynthetic penicillins used in combination with vancomycin might be a safer alternative.

Two recent retrospective cohort studies support the idea that other beta-lactams when used in combination with vancomycin may lead to less nephrotoxicity^30,31^. In one such study of adults with MRSA bacteremia, Zasowski *et al*. compared 129 patients who received vancomycin alone with 229 patients who received vancomycin and cefepime (the latter had to be administered within 72 hours of starting vancomycin and for at least 24 hours)^30^. In the combination therapy group, the median duration of vancomycin received was 5 days (interquartile range [IQR] 4–9 days) and the median duration for cefepime was 3 days (IQR 2–4 days); those in the monotherapy group received a median of 6 days of vancomycin (IQR 4–10 days). There was no difference in nephrotoxicity between the two groups (5.2% vs. 5.4%, *P* = 0.940)^30^.

In another retrospective cohort study, Truong *et al*. compared 47 patients who received vancomycin monotherapy with 63 patients who received vancomycin and a beta-lactam. Of those in the combination group, 34 (54%) received piperacillin-tazobactam, 4 (6.4%) ceftriaxone, 2 (3.2%) ceftaroline, and 2 (3.2%) cefepime while 20 patients (31.8%) received multiple beta-lactams during their treatment course^31^. Combination therapy was started a median of 5 hours (IQR 2–16 hours) from the onset of MRSA bacteremia, and the median duration of beta-lactam therapy was 6 days (IQR 3–9). The authors observed virtually no difference in adverse events (including nephrotoxicity) between the two groups, reporting 11 in the monotherapy group and 12 in the combination group (23.4% vs. 19.1%, *P* = 0.816)^31^. Of note, this is in contrast to increasing evidence from other retrospective trials that suggest that the combination of vancomycin and piperacillin/tazobactam is associated with nephrotoxicity^34,35^. The heterogenous study populations and regimens used in these studies make firm conclusions challenging.

A large randomized controlled trial of vancomycin in combination with a first- or second-generation cephalosporin such as cefazolin could help determine if this regimen is a useful and less toxic alternative for particular patient populations with hard-to-treat MRSA bacteremia. However, this type of study is likely not feasible. Given the current level of evidence, combination vancomycin and beta-lactam regimens should not be the standard of care for MRSA bacteremia and, when used as a salvage regimen, should be combined with efforts to reduce nephrotoxicity, such as optimizing vancomycin dosing and avoiding anti-staphylococcal penicillins.

## Daptomycin combination therapy

Daptomycin dosed at 6 mg/kg per day has been associated with the emergence of resistant MRSA strains and associated treatment failure^36^. Despite the use of higher doses (8–10 mg/kg per day), treatment failure has also been reported^37^. Combination therapy has been explored as a mechanism to prevent the emergence of daptomycin resistance. Daptomycin in combination with beta-lactams is an attractive alternative to vancomycin combination therapy given its favorable toxicity profiles. Retrospective studies suggest that this combination may be a reasonable alternative for salvage therapy in the setting of treatment failure. However, prospective studies addressing this combination have been limited in number.

Geriak *et al*. compared the combination of daptomycin with ceftaroline, a fifth-generation cephalosporin with activity against MRSA, versus vancomycin or daptomycin monotherapy in a small pilot prospective study^29^. Forty patients with MRSA bacteremia from three hospitals were randomly assigned at 72 hours of the initial blood culture to receive either combination therapy of daptomycin 6–8 mg/kg daily and ceftaroline 600 mg every 8 hours (n = 17) or monotherapy (vancomycin, n = 21; daptomycin, n = 2). The primary outcome was a composite of in-hospital mortality and bacteremia duration. The study was stopped early by the investigators because of a mortality difference observed at 90 days: 7 (30%) deaths in the monotherapy group and none in the combination therapy group^29^.

The limitations of this study have been discussed^29,38^. First, the small sample size suggests that the results should be considered only hypothesis-generating and not practice-changing, as was emphasized by the authors. Second, there were important differences between baseline patient characteristics in both groups, most notable of which was that five patients in the monotherapy group had an active malignancy, two of which had end-stage lung cancer, conditions which might have contributed directly to mortality. In contrast, the mean duration of bacteremia did not differ between the two arms, suggesting that factors other than microbiological efficacy contributed to the difference in mortality. Third, as with the CAMERA-2 trial, daptomycin monotherapy was under-represented; only two patients received this regimen. In addition, in the real world, the cost of therapy must be considered. The average daily cost when daptomycin (6–10 mg/kg IV every 24 hours) is combined with ceftaroline (600 mg IV every 12 hours) is about 10 times the cost of standard of care^39^. Some advocate for higher dosing of ceftaroline when used for endovascular infections (600 mg IV every 8 hours), which would further increase costs. Of note, clinical success has been described with both dosing strategies^40–42^.

Some observations from this study are noteworthy. First, dual therapy was limited to a small fraction of the antibiotic course (mean of 8 days), suggesting the possibility that combination therapy is not necessary for the entire course of treatment, which could limit costs. This has also been observed in other small retrospective clinical trials^43^, and de-escalation to a single agent has shown promise in *in vitro* analyses^21^. Second, close to 50% of participants in both groups had a primary endovascular infection as defined by infective endocarditis, cardiac device–associated infections, and vascular/vascular graft infections. This is in contrast to the CAMERA-2 trial, in which this group represented only 10 to 30% of the enrollees. One might hypothesize that patients with primary endovascular infection represent a unique patient population and will particularly benefit from combination therapy^28^; future research focused in this direction would be useful.

The use of daptomycin and ceftaroline combination therapy showed promise in a retrospective matched cohort study. McCreary *et al*. matched MRSA bacteremia patients who received daptomycin plus ceftaroline for at least 72 hours at any point in therapy (n = 58) with those who received vancomycin or daptomycin monotherapy or both (n = 113)^33^. Of those in the monotherapy arm, 96% received vancomycin initially, but 63 (56%) of 113 these patients were switched to an alternative monotherapy regimen, most commonly daptomycin (46/63, 73%), at some point in their course (data not reported)^33^. Combination therapy was used as both initial therapy (within 72 hours of index positive culture) and salvage therapy (51% second, 46% third, and 3% fourth regimen used), leading to the inclusion of a heterogenous population of patients. Switching to combination therapy early was associated with a shorter median duration of bacteremia (5 versus 11.5 days for those switched within 72 hours vs. after 72 hours, respectively; *P* <0.001), but overall mortality was similar between the two groups (8.3% in combination group vs. 14.2% in monotherapy group, *P* >0.05). The interpretation of these results is limited by the heterogeneous patient population and treatment regimens introduced by non-randomized treatment options.

A retrospective study addressing the prohibitive cost associated with the combination of daptomycin and ceftaroline was recently published^32^. Jorgensen *et al*. developed a propensity-matched retrospective cohort study in two hospitals in Detroit and compared MRSA bacteremia patients who received daptomycin monotherapy with those who received daptomycin in combination with a beta-lactam other than ceftaroline (cefepime n = 31, 43% or cefazolin n = 18, 25%)^32^. Patients were included if they received at least 5 days of daptomycin started within 120 hours of the index blood culture and at least 24 hours of a beta-lactam started within a day of daptomycin initiation. The initial choice of therapy was at the discretion of the primary team. The primary outcome was a composite of clinical failure (60-day all-cause mortality) or microbiological failure (recurrent positive blood culture after initial negative)^32^. Patients in the combination therapy group experienced less clinical failure (9 patients [12.5%] vs. 43 patients [27.4%], *P* = 0.013), but only recurrent bacteremia achieved statistical significance. Similar to another study of patients with MSSA bacteremia^44^, AKI was more common in the combination group although chronic renal insufficiency was more frequent in the monotherapy group (10% vs. 2.9%).

Retrospective studies can be challenging to interpret and, in the absence of randomized clinical trials, have an outsized and problematic influence on the standard of clinical care. The lack of randomization in these studies allows for confounders that cannot be fully addressed in the statistical analysis. For example, selection bias and confounding by indication are common problems as the regimens used are heavily influenced by a patient’s clinical presentation and existing institutional policies/guidelines. In addition, the management between and within comparison groups may vary significantly and is incompletely documented (treatment duration, source control, antibiotic dosing, and so on). Ideally, findings from retrospective studies should be tested in a prospective randomized clinical trial before informing standard of care^45^.

Multiple retrospective studies were included in a recent meta-analysis of 1636 patients from nine studies (four are reviewed in this article^30–33^) that compared MRSA bacteremia or endocarditis patients who received vancomycin or daptomycin monotherapy with those who received either drug in combination with a beta-lactam^46^. Clinical failure rates were significantly lower in those who received combination therapy compared with monotherapy (odds ratio [OR] 0.56, CI 0.39–0.79; *P* <0.01) with lower rates of bacteremia relapse (OR 0.63, CI 0.43–0.92; *P* = 0.02) and persistent bacteremia (OR 0.56, CI 0.43–0.75; *P* = 0.01). But again, there was no difference in mortality and nephrotoxicity between the two groups, and the definition of clinical failure rates differed between studies. Some of these outcomes were influenced by the duration of bacteremia. Well-designed clinical trials are necessary to evaluate the efficacy of daptomycin in combination with lower-cost and narrower-spectrum beta-lactams.

## Other combination therapy

There is growing interest in the clinical efficacy of daptomycin in combination with antibacterials other than beta-lactams but limited prospective evidence to inform this practice. One exception is the combination of fosfomycin and daptomycin.

*In vitro* studies demonstrate synergistic activity between daptomycin and fosfomycin, a broad-spectrum bactericidal antibiotic with activity against MRSA^47–49^. A recent open-label, multicenter, randomized clinical trial in patients with MRSA bacteremia and endocarditis compared daptomycin monotherapy (10 mg/kg per day) with the combination of daptomycin and intravenous fosfomycin (2 g every 6 hours)^50^. The protocol directed that the duration of treatment be 10 to 14 days for uncomplicated bacteremia and 28 to 42 days for complicated bacteremia. The primary endpoint was treatment success 6 weeks after the end of therapy.

A total of 82 patients received combination therapy while 85 patients received daptomycin monotherapy. In the modified intention-to-treat population, 74 patients received combination treatment and 81 received standard therapy. Treatment success was attained in 54.1% in the combination arm and 42% in the monotherapy arm (relative risk 1.29, CI 0.93–1.8; *P* = 0.133). No cases of clinical or microbiological failure were observed in the combination group compared with 14.8% in the monotherapy arm (*P* <0.001). At 6 weeks, combination therapy was associated with lower rates of complicated bacteremia (16.2% vs. 32.1%; *P* = 0.022). There was no significant difference in overall mortality between the two groups. The incidence of adverse events leading to treatment discontinuation was higher in the combination group (17.6% vs. 4.9%; *P* = 0.012).

Although there were no significant differences between the two groups, the study population may have represented a less sick population compared with other studies. The median number of days of therapy administered to both groups was 14 days, suggesting that most of the patients in this study had uncomplicated disease, which limits generalizability of this study, especially to those with difficult-to-treat infections for which non-standard therapy is often considered. Another criticism is that this study was not blinded, introducing potential bias. Though available in other countries, the intravenous formulation of fosfomycin is not currently approved for use in the US but it is being reviewed for use in complicated urinary tract infections^51^.

More studies examining non-beta-lactam antibiotics as the second agent are warranted. One such study that is currently enrolling is the CASSETTE trial, an open-label randomized controlled trial that will compare patients with severe *S. aureus* infection who receive standard treatment with those who also receive clindamycin administered for 7 days^52^.

## Persistent bacteremia: does the duration of bacteremia matter?

*S. aureus* is the most common cause of persistent bacteremia (occurring in up to 39%^53^ of cases) and is associated with metastatic infections and relapse^54,55^. The very definition of “persistence” is controversial and multiple variations are used in the literature, ranging from at least 2 days to more than 7 days^56,57^. The 2011 U.S. national guidelines, which are being revised, recommended re-evaluating treatment when bacteremia persists for at least 7 days despite appropriate antibiotic therapy. What further complicates this issue is that the duration of bacteremia is a function of adequate source control, which can be challenging to achieve in clinical practice and is inconsistently documented in clinical trials^58^.

But does duration of bacteremia matter when it comes to relevant clinical outcomes? Combination therapy led to shorter durations of bacteremia in the studies reviewed above, yet there was no difference in mortality in most of these studies. Despite this, two recently published prospective observational studies suggest that duration of bacteremia does matter^59,60^. In a secondary analysis of a multicenter prospective observational cohort study of patients with SAB, 90-day mortality increased if bacteremia persisted for more than 2 days (adjusted hazard ratio 1.93, CI 1.51–2.46; *P* <0.0001)^59^. Of note, only 105 (11%) of the 987 patients in this analysis had an MRSA infection. In a prospective observational study of 884 patients with SAB (290, 33% MRSA), Minejima *et al*. found that 30-day mortality increased with a longer duration of bacteremia^60^. Each additional day of bacteremia was associated with a relative risk of death of 1.16 (CI 1.10–1.22; *P* <0.0001), and a significant increase in mortality risk was seen at 3 days.

Persistent bacteremia often leads to reconsideration of the administered therapy, but failure to detect and remove the focus of infection is the most important driver in most cases. Persistently positive blood cultures should prompt clinicians to search diligently for a persistent source of infection early in the course of therapy. When there is no evidence to suggest an undrained foci of infection, use of an alternative agent may be justified, especially in critically ill patients.

## Conclusions

The optimal treatment of MRSA bacteremia remains unclear. Multiple barriers, including inconsistent case definitions and achieving adequate sample size, prevent the completion of high-quality randomized controlled trials designed to clearly answer challenging clinical questions^45^. Despite the large number of retrospective studies published, the ideal empiric and definitive antibacterial regimens that optimize clinical efficacy and minimize harm remain unknown.

The most critical clinical trials needed in SAB management are those addressing in which patients combination therapy is warranted, whether AUC-guided vancomycin dosing improves patient outcomes, and in which clinical situation agents such as ceftaroline monotherapy, long-acting agents, or oral therapy is appropriate. Finally, future studies should incorporate new statistical methods such as the desirability of outcome ranking (DOOR) approach, which combines both efficacy and toxicity outcomes into one global outcome in order to produce results that are clinically meaningful^61^.
